# Molecular subtype identification and signature construction based on Golgi apparatus-related genes for better prediction prognosis and immunotherapy response in hepatocellular carcinoma

**DOI:** 10.3389/fimmu.2023.1113455

**Published:** 2023-03-27

**Authors:** Liang Sun, Zitao Liu, Zhengyi Wu, Ke Ning, Junwen Hu, Zhendong Chen, Zhipeng Wu, Xiangbao Yin

**Affiliations:** ^1^ Department of General Surgery, The Second Affiliated Hospital of Nanchang University, Nanchang, China; ^2^ Department of Emergency, The First Affiliated Hospital of Nanchang University, Nanchang, China

**Keywords:** golgi apparatus, hepatocellular carcinoma, signature, prognosis, immunotherapy

## Abstract

**Introduction:**

The Golgi apparatus (GA) is the center of protein and lipid synthesis and modification in normal cells and is involved in regulating various cellular process as a signaling hub, the dysfunction of which can lead to the development of various pathological conditions, including tumors. Mutations in Golgi apparatus-related genes (GARGs) are prevalent in most tumors, and their mutations can make them pro-tumor metastatic. The aim of this study was to analyze the predictive role of GARGs in the prognosis and immunotherapeutic outcome of hepatocellular carcinoma.

**Methods:**

We used TCGA, GEO and ICGC databases to classify hepatocellular carcinoma samples into two molecular subtypes based on the expression of GARGs. Signature construction was then performed using GARGs, and signature genes were selected for expression validation and tumor phenotype experiments to determine the role of GARGs in the prognosis of hepatocellular carcinoma.

**Results:**

Using the TCGA, GEO and ICGC databases, two major subtypes of molecular heterogeneity among hepatocellular carcinoma tumors were identified based on the expression of GARGs, C1 as a high-risk subtype (low survival) and C2 as a low-risk subtype (high survival). The high-risk subtype had lower StromalScore, ImmuneScore, ESTIMATEScore and higher TumorPurity, indicating poorer treatment outcome for ICI. Meanwhile, we constructed a new risk assessment profile for hepatocellular carcinoma based on GARGs, and we found that the high-risk group had a worse prognosis, a higher risk of immune escape, and a higher TP53 mutation rate. Meanwhile, TME analysis showed higher tumor purity TumorPurity and lower ESTIMATEScore, ImmuneScore and StromalScore in the high-risk group. We also found that the high-risk group responded more strongly to a variety of anticancer drugs, which is useful for guiding clinical drug use. Meanwhile, the expression of BSG was experimentally found to be associated with poor prognosis of HCC. After interfering with the expression of BSG in HCC cells SMMC-7721, the proliferation and migration ability of HCC cells were significantly restricted.

**Discussion:**

The signature we constructed using GARGs can well predict the prognosis and immunotherapy effect of hepatocellular carcinoma, providing new ideas and strategies for the treatment of hepatocellular carcinoma.

## 1 Introduction

The treatment of hepatocellular carcinoma (HCC) is currently a major challenge worldwide, and despite the availability of multiple treatment options, treatment outcomes continue to fall short of expectations. Therefore, new breakthroughs and tools are needed to address the current treatment bottlenecks.

The Golgi apparatus (GA) has long served as a platform for sorting, modification, production and transport of proteins and lipids. In addition to these classical functions, studies have confirmed that the GA plays an important role in the regulation of many cellular processes, such as cell migration, apoptosis, inflammation, autophagy, and stress responses (1). The mechanism of action of intracellular GA is a finely regulated process, and dysregulation can lead to a variety of pathological conditions. Increasing evidence suggests that GA functions are closely related to cancer development and progression, including the regulation of cell proliferation, motility, metabolism and immune evasion (2, 3). The GA in cancer cells frequently exhibits functional and structural disorders associated with cancer development and progression (4, 5). In addition, the GA can also influence cellular metabolism and participate in processes that control the phenotype of cancer cells, including innate immune response, angiogenesis, tumor migration and invasion (6, 7). Moreover, Golgi apparatus-related genes (GARGs) have been found to be mutated at a high frequency in tumors, and mutated GARGs tends to enhance tumor metastasis and bring a poorer prognosis (8). It has also been shown that the chromosome 1q21-43 region (containing many Golgi-related functional genes) is frequently amplified in multiple cancer types (8).

The role of GARGs in tumors has been confirmed. Among them, GOLM1 can regulate EGFR/RTK and thus promote metastasis of HCC (9). In addition, GOLM1 can control colitis and colon tumorigenesis by regulating the balance of Notch signaling in the intestine (10). Golgi phosphoprotein 3 (GOLPH3) is involved in cell migration, GA morphology and orientation, and protein glycosylation, and the increased expression of GOLPH3 observed in a variety of tumors enhances cis-transport of GA and leads to increased cytosolic ejection of pro-metastatic factors such as cytokines, growth factors, and Wnt molecules (11, 12). In addition, GA can enhance the secretion of immune factors and promote the formation of an immunosuppressive tumor microenvironment that promotes tumor progression and metastasis. For example, PI4KIIβ promotes the migration of myeloid-derived suppressor cells, a key immunosuppressive cell type associated with metastasis, by mediating the cytokine of immune modifying molecules (CXCL1, IL-1α, IL-8 and VEGF) in lung adenocarcinoma cells (13). GOLPH3 interacts with cytoskeleton-associated protein 4 localized in the extracellular body and is able to enhance the secretion of Wnt3a, thereby limiting T cell differentiation (14).

Therefore, GARGs play an important role in tumor development and immune effects, which is a new direction to be explored. In this study, a prognostic and immune response system based on GARGs for hepatocellular carcinoma was constructed by bioinformatics analysis using public databases to provide guidance for clinical treatment of hepatocellular carcinoma.

## 2 Materials and methods

### 2.1 Data processing

Search and download the gene set “GOCC_GOLGI_APPARATUS” through Gene Set Enrichment Analysis (GSEA) MSigDB database (http://www.gsea-msigdb.org/gsea/msigdb/index.jsp).

The mRNA expression data and clinical information of samples from patients with HCC were downloaded from the TCGA, GEO and ICGC databases, of which the TCGA database included 374 HCC samples and 50 normal liver samples, and 365 HCC samples were retained after screening (excluding HCC samples with survival time of 0). the GEO database (GSE76427 and GSE14520) obtained data for 115 and 219 HCC samples, respectively. Thus, TCGA-LIHC, GSE76427 and GSE14520 had a total of 699 HCC samples. the ICGC database (ICGC-LIRI-JP) excluded blood samples and samples with metastatic tumors, and finally retained data for 208 HCC samples.

### 2.2 Gene screening

Valuable GARGs were screened for further analysis using the TCGA database. First, we used the weighted gene co-expression network analysis (WGCNA) package to screen out the most relevant modules for HCC, and extracted the genes from the modules for subsequent analysis. Second, differential expression analysis was performed for normal and tumor samples of TCGA. We used the “limma” R package and Wilcoxon test to screen Golgi apparatus-related differentially expressed genes between normal and HCC samples in the TCGA cohort (FDR< 0.001, |logFC|> 1). Third, COX analysis of GARGs was performed to obtain prognosis-related genes. Finally, the overlapping genes of the three were screened for the next step of analysis.

### 2.3 Identification of GARGs molecular subtypes by consensus clustering

First, we merged the HCC sample data from the three datasets TCGA-LIHC, GSE76427 and GSE14520. TCGA-LIHC data (FPKM normalized) were converted to Transcripts Per Kilobase per Million mapped reads (TPM). For the microarray cohort, the normalized matrix files of expression data and clinical information were downloaded directly and log-transformed, GSE76427 and GSE14520 using the “sva” R package to eliminate batch effects (15). Then batch correct and normalize the three data sets (**Figures S1A, B**).

Then, we used the R package “ConsensusClusterPlus” to perform consensus clustering analysis on the screened overlapping gene-related molecular subtypes. Next, we used the R packages “survival” and “survminer” to investigate the relationship between subtypes and OS. Principal component analysis was used to identify whether the two subtypes were able to distinguish well between HCC samples. The R package “pheatmap” was used to show the relationship between molecular subtypes of GARGs and clinicopathological characteristics (sex, age and Stage).

### 2.4 Gene set variation analysis and single sample gene set enrichment analysis

Gene set variation analysis (GSVA) was performed using the KEGG gene set (c2.cp.kegg.v7.5.1.symbols) to assess pathway differences across molecular isoforms. Biosignature gene sets were obtained from the Hallmarker gene set. Single sample gene set enrichment analysis (ssGSEA) was used to assess the relative activity of biological pathways between different isoforms.

### 2.5 Analysis of tumor microenvironment among molecular subtypes

TME is a dynamic system that undergoes specific modifications during the development of cancer and eventually leads to metastasis and diffusion, which is still the main cause of death of cancer patients (16). Tumorigenesis is a complex and dynamic process. TME includes intercellular compartments composed of stromal fibroblasts, infiltrating immune cells, blood and lymphatic networks, and non-cellular components including ECM (17). We analyzed the characteristics of the two molecular subtypes by comparing the content of non-tumor cell components and tumor purity between the two molecular subtypes. Understanding the interactions between different immune cells and between immune cells and stromal cells (such as hematopoietic stem cells or CAFs) will be crucial to the therapeutic development of tumor microenvironment.

### 2.6 Construct and verification of GARGs signature

To further represent the prognostic value of GARGs in HCC, we constructed a risk score system constructed from GARGs using the TCGA database and GEO database to predict prognosis and immunotherapeutic response in hepatocytes. We constructed a signature using Cox regression analysis and lasso penalty method using previous overlapping genes to obtain a risk scoring system based on GARGs: $riskScore=\;\sum_{i=1}^{n}(coef_{i}\;*\;exp_{i})$ , The samples were divided into two groups (high-risk and low-risk) based on the median value of risk scores, and then survival analysis, principal component analysis (PCA), and ROC curve plotting were performed, followed by nomograms to further determine the prognosis of patients. Also, to verify the reliability of the signature, we validated the signature using an external dataset ICGC database to determine its accuracy and stability.

### 2.7 Quantitative real-time PCR

After obtaining informed consent from patients, we collected 20 pairs of HCC tissues and paraneoplastic tissues (from the Second Affiliated Hospital of Nanchang University), then cultured one normal hepatocyte line (HL-7702) and four HCC cell lines (HCCLM3, MHCC97H, HepG2 and SMMC-7721), and after extracting RNA from the tissues and cells, q-PCR experiments were performed to verify the signature genes.

### 2.8 Immunohistochemistry experiments

Immunohistochemistry (IHC) experiments was used to detect protein expression differences: HCC tissues were paraffin-embedded, sectioned, dewaxed and hydrated, incubated with anti-trait gene antibodies overnight at room temperature, then labeled with secondary antibodies for 30 minutes, stained and photographed.

### 2.9 Immunofluorescence

We inoculated 2000 SMMC-7721 cells onto a 24-well plate. After 24-36 hours, wash them with PBS three times, and then fix them with 4% paraformaldehyde for 20 minutes. At room temperature, permeate the plate with 0.5% Triton X-100 for 20 minutes, and then seal it with goat serum. We used BSG antibody (1:100, Abbart, T59564), KIF20A antibody (1:200, Abbart, PU602304), RNF24 antibody (1:1000, Abbart, PH9881) and GM130 antibody (1:2000, Protentech, 11308-1-AP) to incubate overnight. The labeled antibodies of different colors were incubated at room temperature for 1 hour, and then re-stained with DAPI. Finally, fluorescence microscope is used for imaging.

### 2.10 Cell proliferation assay

EdU assay: Inoculate SMMC-7721 cells with 1*10^4^/well in 96-well plate, and after 24 hours, Each well was incubated with 100 ul of 50 uM EdU (YF ^®^ 594 Click-iT EdU imaging kit, UELANDY) for 2 hours, and then fixed with 4% paraformaldehyde for 30 minutes. Use 2 mg/ml glycine for another 5 minutes. After washing with PBS twice, the cells were mixed with 1 × The Apollo staining solution was incubated for 30 minutes. Discard the dye solution and wash twice with PBS containing 0.5% TritonX-100. Join 1 × Hoechest 33342 was incubated at room temperature for 30 minutes. After washing with PBS, observe the positive cells by fluorescence microscope.

### 2.11 Wound healing assay

Inoculate cells into six orifice plates and use 200 μl disposable gun head forms wound line. When the cell grows to about 80% - 90%, draw lines on the cell surface. Wash the plate twice with PBS to remove suspended cells. The wound closure images were taken at 0, 24 and 48 hours.

### 2.12 Transwell assay

Cells were inoculated into the upper cavity of 200ul serum-free medium (1.5*10^4^/well). Add 600ul of medium containing 10% serum into the lower chamber. After 24 hours, the cells were fixed and stained with crystal violet, and then the migrated cells were counted under the inverted optical microscope.

### 2.13 Immune infiltration analysis and immune function analysis

Correlations between signature genes and 22 immune cells were analyzed using the CIBERSORT algorithm, while differences in the abundance of immune cells between high and low risk groups were analyzed to distinguish features between the two risk groups.

### 2.14 Immune escape and drug sensitivity analysis

We evaluated the Tumor Immune Dysfunction and Exclusion (TIDE) scores between the risk groups to determine whether there were differences in the effects of immunotherapy between the risk groups. The IC50 values of chemotherapeutic drugs were also calculated by the “PRROPHIC” R package to assess the sensitivity of the different risk groups to chemotherapeutic drugs.

### 2.15 Statistical analysis

R language (Version 4.1.2) and GraphPad Prism 8.0 are used for statistical analysis. The t-test was used for two groups of analysis. P<0.05 means statistically significant.

## 3 Results

### 3.1 WGCNA

**Figure 1:**
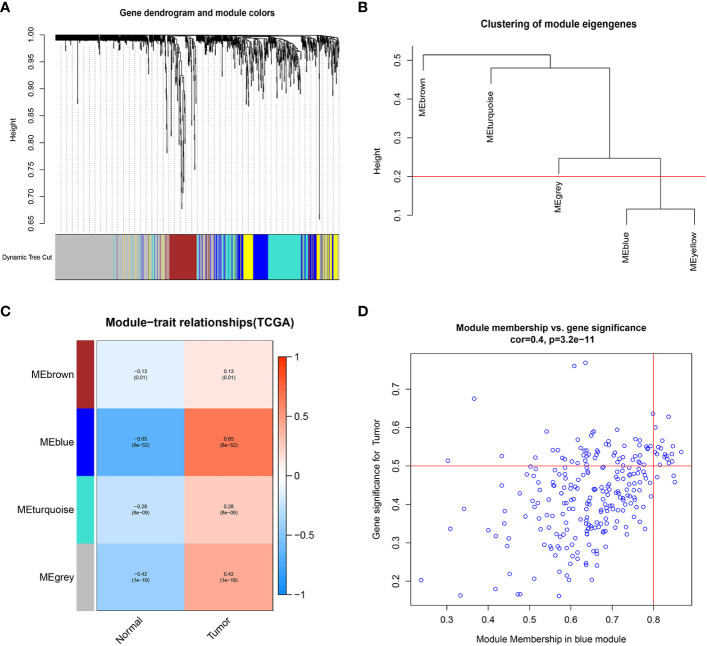
Weighted gene co-expression network analysis. **(A)** Clustering dendrogram of genes, with dissimilarity based on topological overlap, together with assigned module colors. **(B)** Visual representation of the weight relationship of modular genes in an eigengene network. **(C)** Module-trait associations: Each row corresponds to a module eigengene and the column to the tumor and normal. Each cell contains the corresponding correlation and p-value. **(D)** Correlation graph of genes and tumors in the blue module.

We extracted 1659 GARGs from the GSEA database (GOCC_GOLGI_APPARATUS). we used TCGA-LIHC data for differential module analysis by WGCNA algorithm to screen out key genes closely related to HCC, and finally obtained four modules, among which the Meblue module was the most correlated and significantly different, so GARGs in the Meblue module was selected for the subsequent analysis (**Figure 1**).

### 3.2 Differential expression and prognostic of GARGs in TCGA-LIHC

**Figure 2:**
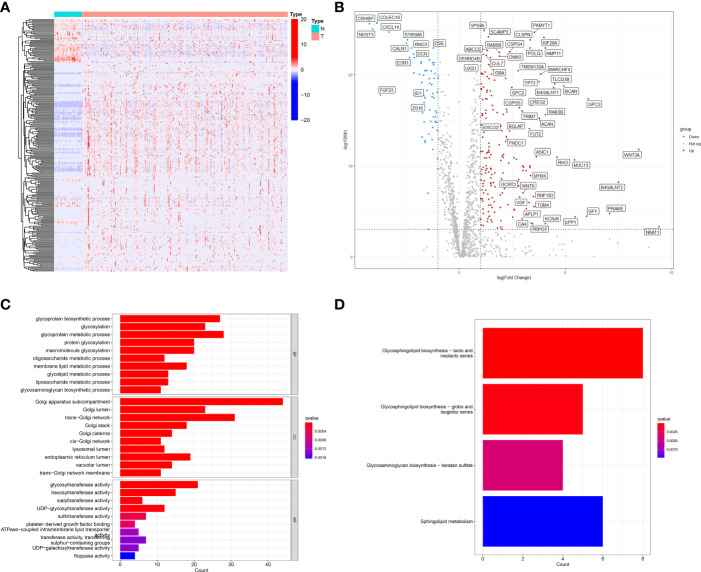
Identification of differentially expressed GARGs in HCC. **(A)** The heatmap. **(B)** The volcano map. **(C)** Gene ontology (GO) analysis of differentially expressed GARGs. **(D)** Kyoto Encyclopedia of Genes and Genomes (KEGG) analysis of differentially expressed GARGs.

First, using TCGA-LIHC data, we screened 50 normal liver tissues and 374 HCC tissues for differentially expressed genes (DEGs) (|logFC|>1, FDR<0.001), and obtained a total of 242 differentially expressed genes (62 down-regulated and 180 up-regulated) (**Table S1**; **Figures 2A, B**). GO and KEGG enrichment analyses were performed on these DEGs. the GO enrichment results showed that GARGs were associated with Golgi apparatus subcompartment, trans-Golgi network, Golgi lumen, Golgi stack and cis-Golgi network. KEGG showed that DEGs were mainly associated with Glycosphingolipid biosynthesis-lacto and neolacto series, Glycosphingolipid biosynthesis-globo and isoglobo series, Glycosaminoglycan biosynthesis-keratan sulfate and Sphingolipid metabolism (**Figures 2C, D**). Then, 672 GARGs associated with prognosis were screened using a one-way COX analysis (**Table S2**).

### 3.3 Screening for core genes

**Figure 3:**
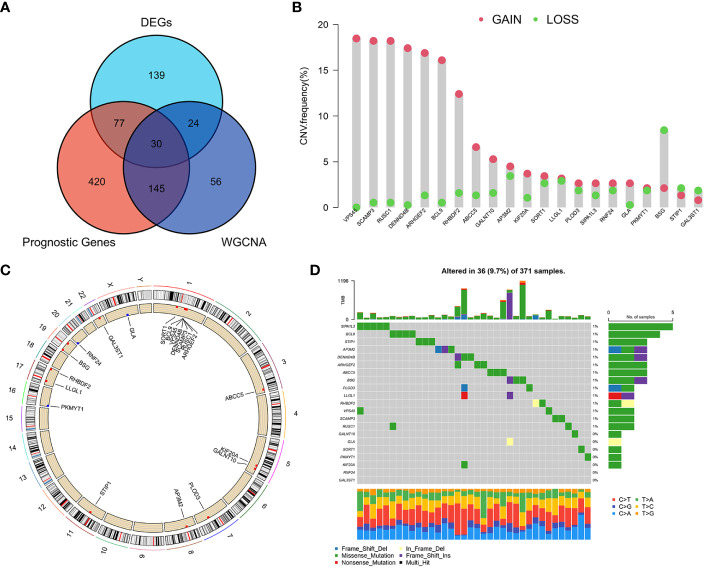
Screening of major GARGs and their Copy Number Variation and Mutations. **(A)** DEGs, prognostic genes and Meblue module genes were intersected by a Venn diagram. **(B)** The CNV variation frequency of GARGs in TCGA-LIHC. **(C)** The location of CNV alteration of GARGs on 23 chromosomes in TCGA-LIHC. **(D)** Mutation frequency of GARGs in TCGA-LIHC.

Based on the WGCNA algorithm, differential expression analysis and prognostic analysis, three groups of genes were obtained for screening, respectively, and we overlapped the three to screen 30 GARGs for subsequent analysis (**Figure 3A**). We extracted the expression data of these 30 genes from the combined mRNA expression data of TCGA-LIHC, GSE76427 and GSE14520, and finally 21 genes were extracted (some genes were not included in all three datasets at the same time) (**Table S3**). Then, the mutation status and copy number changes of these 21 genes were analyzed using TCGA database information (**Figures 3B–D**).

### 3.4 Identification of GARGs molecular subtypes

**Figure 4:**
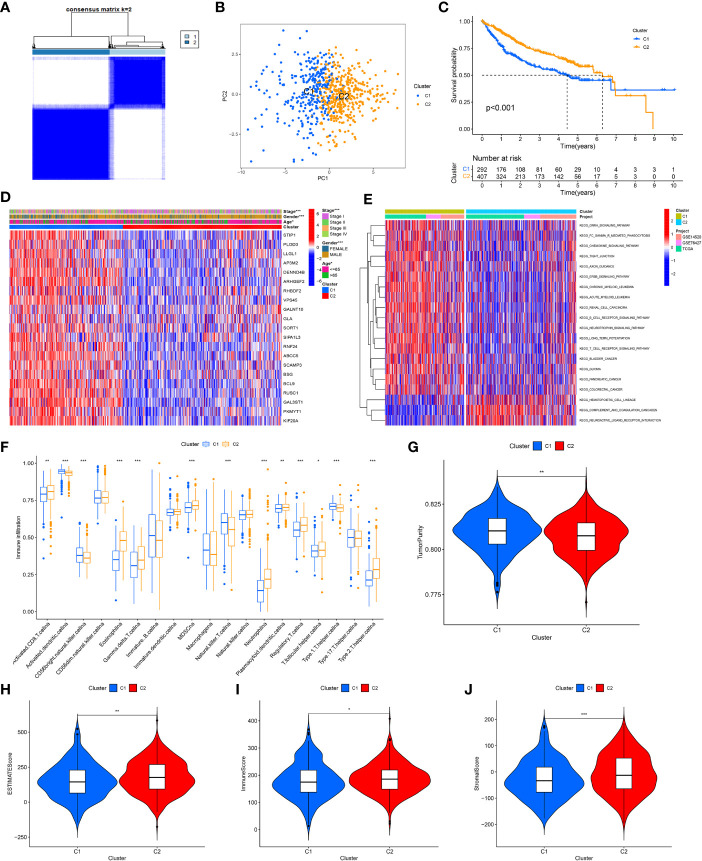
Identification of tumor subtypes based on the GARGs. **(A)** Two clusters were identified according to the best consensus matrix (k=2). **(B)** Principal Component Analysis (PCA) demonstrates the degree of differentiation between the two subtypes. **(C)** The overall survival between the two subtypes. **(D)** Comparison of GARGs expression and clinical characteristics between the two subtypes. **(E)** Gene Set Variation Analysis (GSVA) was performed to analyze the differences between the two subtypes. **(F)** The immune cell infiltration between the two subtypes was analyzed by the ssGSEA. **(G)** TumorPurity between the two subtypes. **(H)** ESTIMATEScore between the two subtypes. **(I)** ImmuneScore between the two subtypes. **(J)** StromalScore between the two subtypes. *P<0.05, **P<0.01, ***P<0.001.

After combining the information of three data sets, based on the expression amount of these 21 genes, we determined two different HCC subtypes (C1 and C2) through consensus cluster analysis, and the two subtypes can be well distinguished (**Figures 4A, B**). The survival analysis shows that the survival rate of patients with C1 is worse (**Figure 4C**), so we call cluster C1 “high-risk type” and cluster C2 “low-risk type”. The expression of 21 GARGs was significantly different in the two subtypes, and there were significant differences in tumor stage, sex and age between the two subtypes (P<0.05) (**Figure 4D**).

To further investigate the biological behavior between these two isoforms, the differences between the two isoforms were analyzed using Gene Set Variation Analysis (GSVA) and the “limma” package in 20 pathways, where the low-risk group was mainly expressed in KEGG_ HEMATOPOIETIC_ CELL_ LINEAG, KEGG_ COMPLEMENT_ AND_ COAGULATION_ CASCADES and KEGG_ NEUROACTIVE_ LIGAND_ RECEPTOR_ INTERACTION (**Figure 4E**). Meanwhile, the immune cell infiltration between the two subtypes was analyzed by the ssGSEA, and the results showed that cluster C2 had significantly higher immune infiltration in Activated CD8 T cell, Eosinophilna, Gamma delta T cell, MDSCna, Neutrophila, Plasmacytoid dendritic cell, Regulatory T cell, T follicular helper cell and Type 2 T helper cell. In contrast, immune infiltration was higher in Activated dendritic cellna, CD56bright natural killer cellna, Natural killer T cellna and Type 1 T helper cellna in cluster C1 (**Figure 4F**).

To further typify the characteristics and tumor microenvironment between the two subtypes, we evaluated ImmuneScore, StromalScore, ESTIMATEScore and TumorPurity between the two subtypes using the ESTIMATE method, which showed that the high-risk group had higher tumor purity and lower immune and stromal cell content (**Figures 4G–J**).

### 3.5 Construction of a prognostic signature based on GARGs

**Figure 5:**
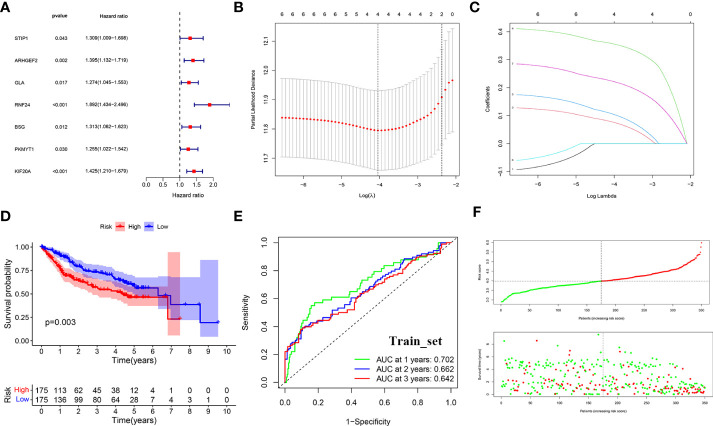
LASSO-COX regression to identify signature genes in the TCGA-Train set. **(A)** Forest plot of prognostic GARGs in the train set. **(B)** Cross-validation of the LASSO regression. **(C)** Coefficient value of prognostic genes. **(D)** Survival analysis between the high and low risk groups. **(E)** Receiver operating characteristic curve (ROC) of risk score. **(F)** Distribution of risk scores and survival outcomes.


To further analyze the value of GARGs in the prognosis and immune response of HCC, we constructed a prognostic signature of HCC based on these 21 genes. First, 699 HCC samples were randomly divided into two groups (training set (set1) and test set (set2)) by 1:1, and the total samples were used as test set (set3). A prognostic signature consisting of three GARGs was built by LASSO-COX analysis in the training set (**Figures 5A–C**).

riskScore = BSG expression * 0.1756 + KIF20A expression* 0.2711 + RNF24 expression * 0.3953

The risk score was calculated for each patient according to the formula. The median was used to divide the sample into high-risk and low-risk groups, and in the training set, survival was significantly lower in the high-risk group (p<0.001) (**Figure 5D**). the ROC curve assessed the accuracy of the signature, with AUC values of 0.702, 0.662, and 0.642 at 1, 2, and 3 years, respectively (**Figure 5E**). And as the risk score increased, the survival time gradually decreased and the number of deaths gradually increased (**Figure 5F**).

### 3.6 Validation of prognostic signature

**Figure 6:**
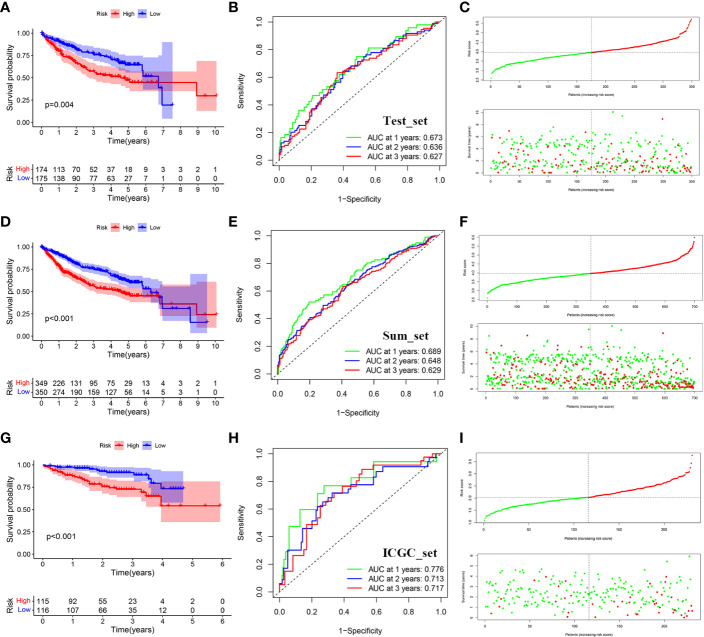
Distribution of survival analysis, ROC and risk scores and survival outcomes for the validation set. **(A)** Survival analysis between the high and low risk groups in TCGA-test. **(B)** Receiver operating characteristic curve (ROC) of risk score in TCGA-test. **(C)** Distribution of risk scores and survival outcomes in TCGA-test. **(D)** Survival analysis between the high and low risk groups in TCGA-sum. **(E)** Receiver operating characteristic curve (ROC) of risk score in TCGA-sum. **(F)** Distribution of risk scores and survival outcomes in TCGA-sum. **(G)** Survival analysis between the high and low risk groups in ICGC. **(H)** Receiver operating characteristic curve (ROC) of risk score in ICGC. **(I)** Distribution of risk scores and survival outcomes in ICGC.

We use the test set (set2) and the total data set (set3) to test the accuracy of the model. Similarly, after calculating the risk score of each sample, we can divide it into high and low risk groups according to the median value. The results of KM survival analysis showed that the survival rates of the high-risk groups in set2 and set3 were lower (P<0.05) (**Figures 6A, D**). AUC values assessed by ROC curve in set2 are 0.673, 0.636 and 0.627 respectively in 1, 2 and 3 years (**Figure 6B**). AUC values assessed by ROC curve in set3 are 0.689, 0.648 and 0.629 respectively in 1, 2 and 3 years (**Figure 6E**). In addition, the number of deaths gradually increased with the increase of risk score (**Figures 6C, F**).

To further verify the accuracy of the signature, we use the data of ICGC-LIRI-JP queue as external verification data to verify the risk characteristics. Similarly, the cohort was divided into high-risk group and low-risk group according to the median risk score. The results showed that patients with higher risk also had worse survival outcomes (P<0.05) (**Figure 6G**). AUC values assessed by ROC curve in set3 are 0.776, 0.713 and 0.717 respectively in 1, 2 and 3 years (**Figure 6H**). Similarly, as the risk score went up, the number of deaths gradually increased (**Figure 6I**). Meanwhile, we used IMvigor database to verify that there was a significant difference between the immunotherapy response group and the non-response group (**Figure S2**).

Also, the results of the t-distributed Stochastic Neighbor Embedding (t-SNE) and Principal Component Analysis (PCA) analyses demonstrate the discriminative power of the signature (**Figure S3**).

### 3.7 Expression validation and epigenetic analysis of signature genes

**Figure 7:**
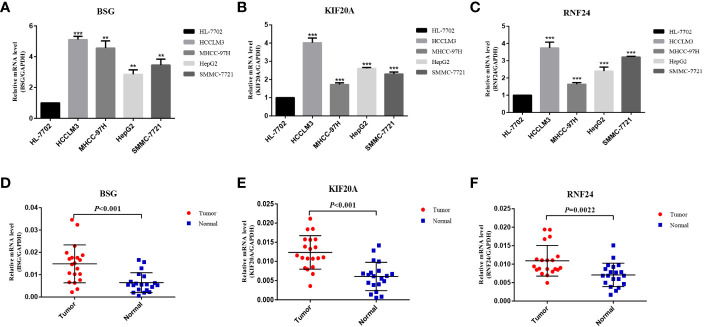
Validation of differential expression of 3 signature genes in cells and tissues by q-PCR. **(A–C)** Differential mRNA expression of 3 signature genes in HL-7702, HCCLM3, MHCC-97H, HepG2 and SMMC-7721 cells. **(D–F)** Differential mRNA expression of 3 signature genes in HCC tissue and paraneoplastic tissue (20 pairs). **<0.01, *** <0.001.

**Figure 8:**
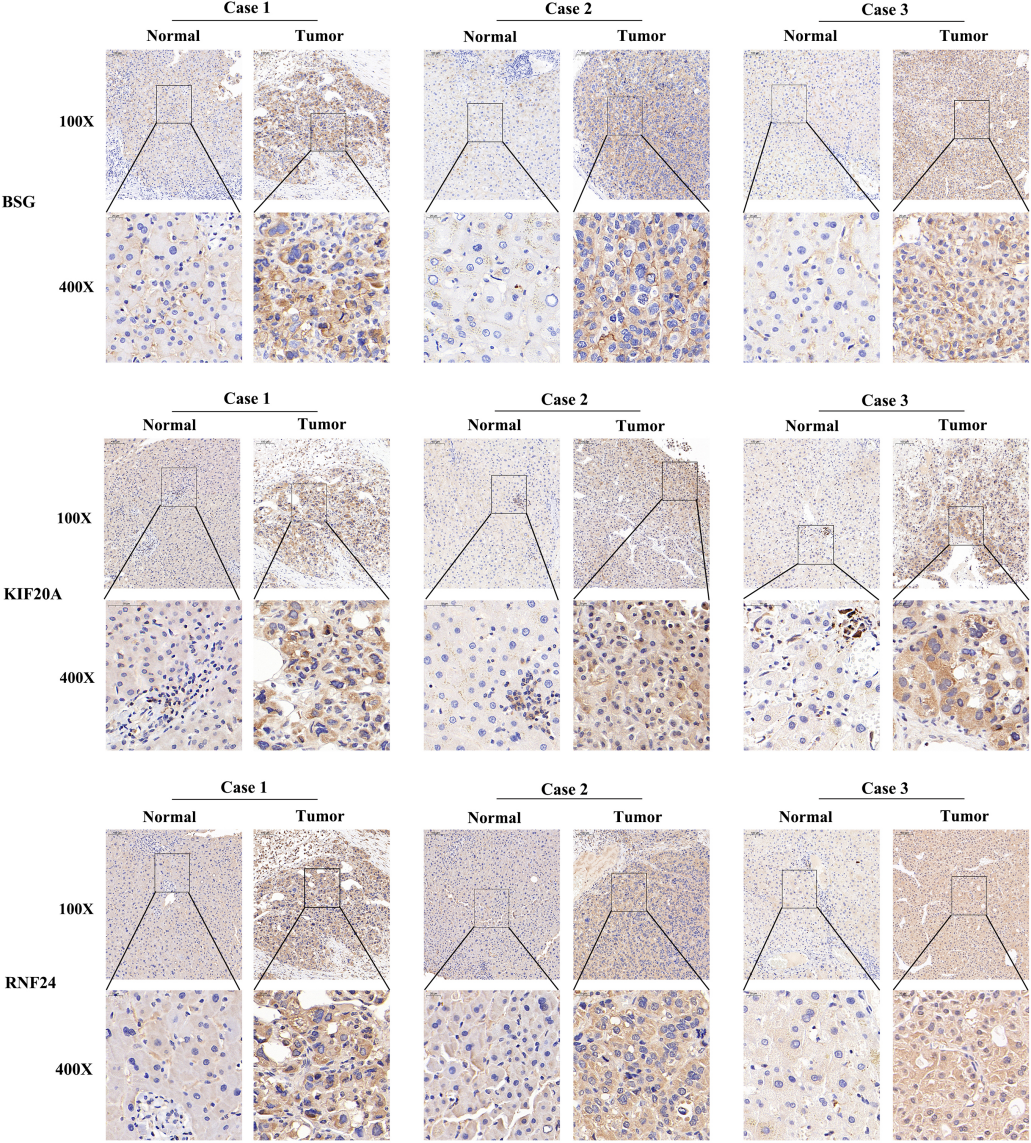
Immunohistochemistry of 3 signature genes in three pairs of HCC tissues and paraneoplastic tissues.

At the RNA level, we verified the expression of the signature genes in normal hepatocytes (7702) and hepatocellular carcinoma cells (HCCLM3, MHCC97H, HepG2 and SMMC-7721) by q-PCR assay (**Figures 7A–C**). In addition, we collected 20 pairs of HCC tissues and their paraneoplastic tissues to detect the mRNA expression of the signature genes (**Figures 7D–F**), and the primer sequences are shown in **Table 1**. At the protein level, we detected the difference in the expression of the signature genes in three pairs of HCC tissues and paraneoplastic tissues by immunohistochemical assay, and the results were consistent with the results at the RNA level, and the signature genes were expressed in HCC tissues higher (**Figure 8**).

**Table 1:** Primer sequences used for RT-qPCR.

Gene	Sequence (5’-3’)
GAPDH	F: GGAGCGAGATCCCTCCAAAAT
	R: GCTGTTGTCATACTTCTCATGG
BSG	F: GCCGGTCAGAGCTACACATT
	R: GATGATGGCCTGGTCGGAG
KIF20A	F: AGTCACAGCATCTTCTCAATCAG
	R: TTCAACCGTTCACCACTCTTC
RNF24	F: GGCTAAGACATCAAGCACACA
	R: TCTGTGGAAGGCGTGCTTAC

**Figure 9:**
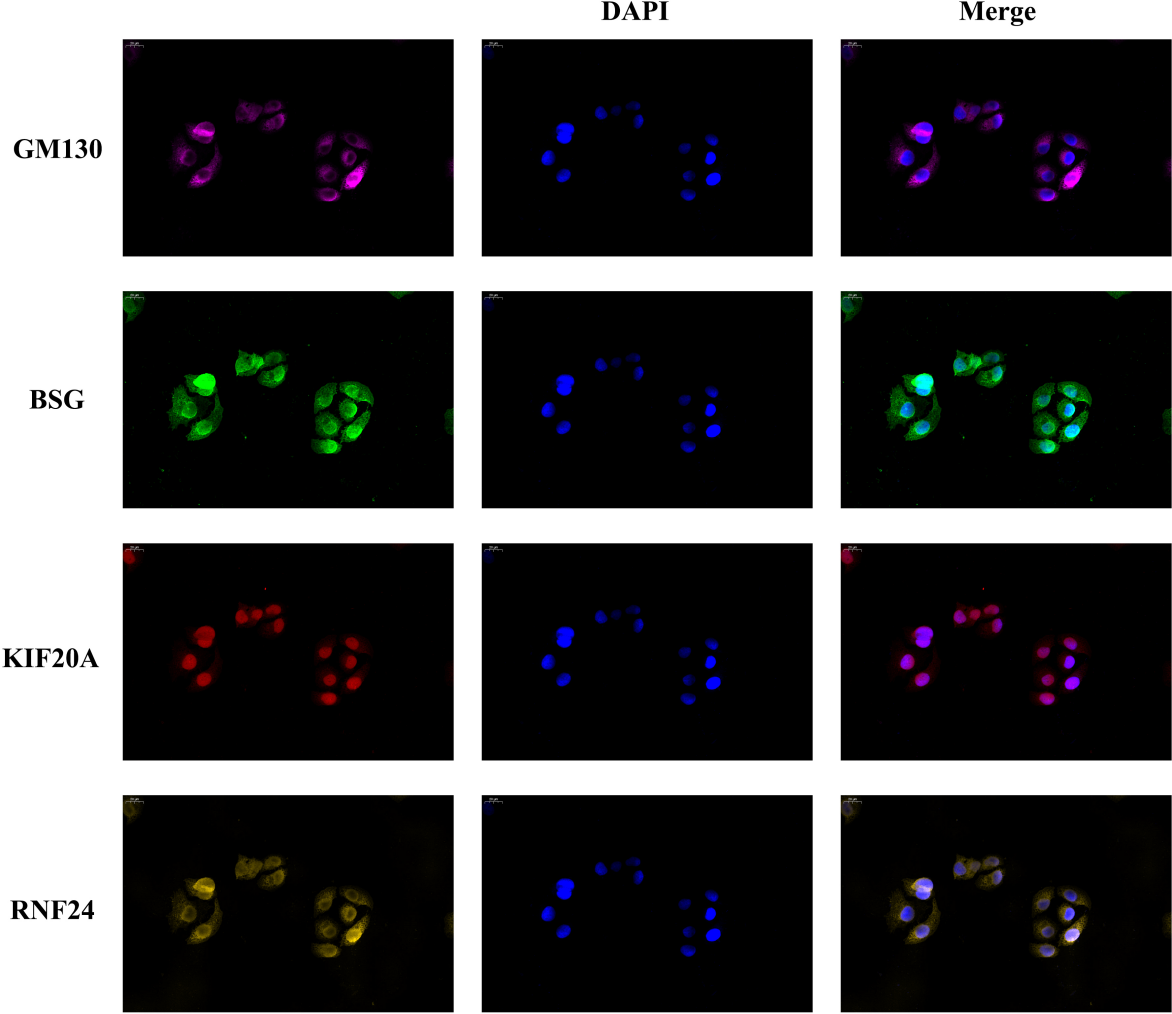
Golgi apparatus marker gene GM130 was used as a reference. Immunofluorescence experiments determined the localization of the signature genes in SMMC-7721 cell.

Since the signature genes are closely linked to the function of the Golgi apparatus, to further verify the localization of these genes, we performed immunofluorescence experiments. We used the Golgi apparatus marker gene GM130 as a control, and the results showed that GM130 was localized in the cytoplasm and the localization of the three signature genes were also predominantly present in the cytoplasm, consistent with the results of our analysis (**Figure 9**).

We analyzed the methylation levels and copy number variation of the signature genes in LIHC using the GSCA database. The results showed that the higher the expression of the three genes, the lower the level of methylation. Meanwhile, the copy number variation and survival of the signature genes were presented (**Figure S4**).

### 3.8 Independent prognostic analysis and construction of nomograms

**Figure 10:**
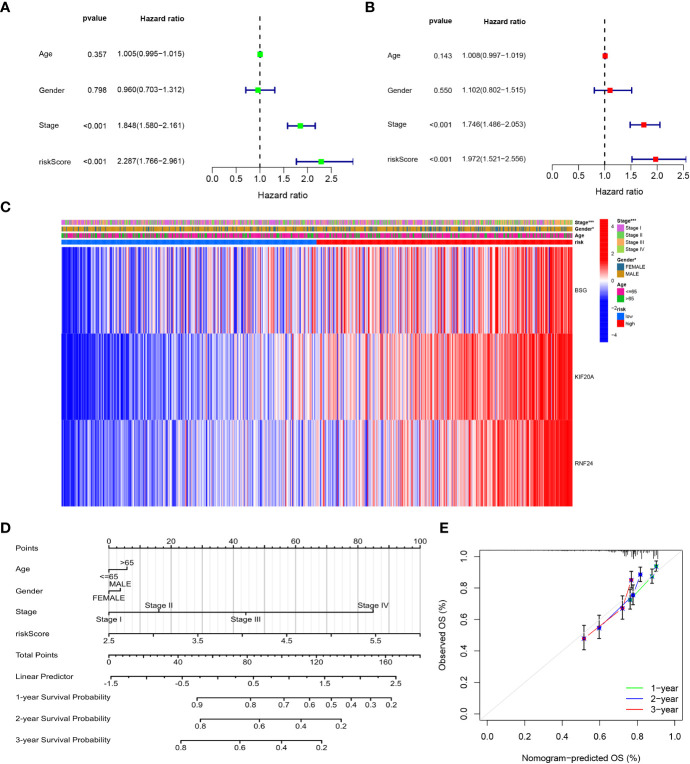
Independent prognostic analysis and Construction and validation of the Nomograms. **(A)** Univariate cox regression analysis for the TCGA cohort. **(B)** Multivariate cox regression analysis for the TCGA cohort. **(C)** The correlations between the GARGs and clinicopathologic characters of the high-risk group and low-risk group were shown as a heatmap. **(D)** Construction of the Nomograms. **(E)** The calibration curves displayed the accuracy of the nomogram in the 1-, 2- and 3 years.

We performed univariate and multivariate analyses of risk scores and clinical data, which showed that clinical stage and risk scores were significantly associated with patient prognosis (P<0.05) in both univariate and multivariate analyses (**Figures 10A, B**). Then, we presented the signature genes with clinical characteristics as heat maps and found significant differences in Stage staging and gender between high- and low-risk groups (P<0.05) (**Figure 10C**). To further evaluate individual patients, we simplified the statistical prediction signature with Nomograms (**Figure 10D**). calibration plots of survival probabilities at 1, 2, and 3 years also showed good agreement between bar graph predictions and actual observations (**Figure 10E**).

### 3.9 BSG expression was associated with poor prognosis of HCC

**Figure 11:**
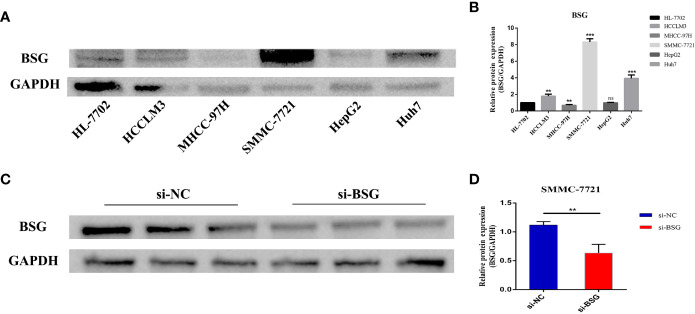
Evaluate the expression level of BSG in HCC cell line, and then verify the intervention efficiency. **(A)** Compared with HL-7702(normal liver cell), BSG is generally highly expressed in tumor cells and has the highest expression in SMMC7721. **(B)** Differential protein expression of BSG between hepatocytes and hepatoma cells. **(C)** Verification of intervention efficiency through western blot. **(D)** The expression of BSG protein was significantly reduced in SMMC-7721 cells after the intervention of BSG expression. **P value<0.01; ***P value<0.001; ns, Not Significant.

We selected the signature gene BSG for the prognosis experiment of HCC. First, we evaluated the expression level of BSG in HCC cell lines. The results showed that BSG was highly expressed in HCCLM3, SMMC-7721 and Huh7 than HL-7702 (normal liver cell) (**Figures 11A, B**). Next, we used SMMC-7721, which has the highest level of BSG mRNA expression. Then, We used Western blot to verify the knockdown efficiency (**Figures 11C, D**).

**Figure 12:**
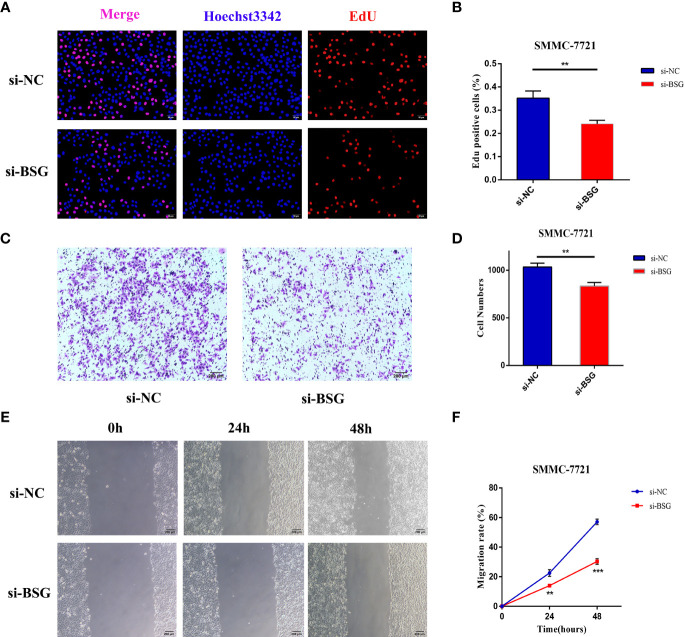
The expression level of BSG is related to the poor prognosis of HCC. **(A, B)** EdU assay showed that the cell proliferation rate of si-BSG group was significantly lower than that of si-NC group. **(C, D)** Transwell assay showed that inhibiting the expression of BSG could significantly inhibit the migration ability of HCC cells. **(E, F)** The wound healing assay showed that the migration ability of HCC cells were significantly inhibited after the intervention of BSG expression. **<0.01, *** <0.001.

To assess the effect of BSG on proliferation in HCC, we used EdU assays in SMMC-7721. The results showed that after interfering with the expression of BSG in SMMC7721 cell, the cell proliferation rate in the si-BSG group was significantly lower than that of si-NC group (**Figures 12A, B**). At the same time, the effect of inhibiting BSG expression on the migration of HCC cells was further analyzed. Transwell assay and wound healing assay showed that inhibition the expression of BSG could significantly inhibit the migration ability of HCC cells (**Figures 12C–F**).

### 3.10 Immune cell infiltration analysis

**Figure 13:**
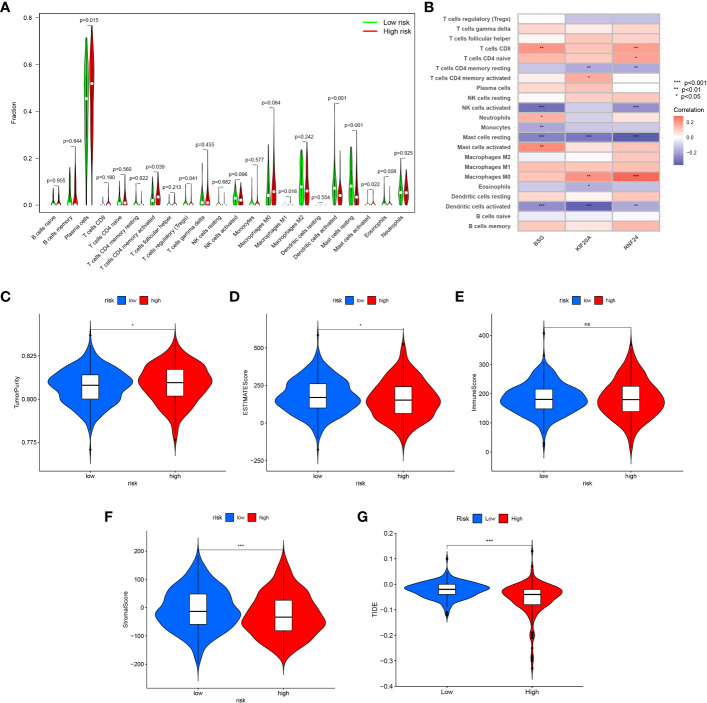
Immune infiltration and tumor microenvironment analysis between high-risk and low-risk groups. **(A)** The CIBERSORT algorithm was used to calculate the immune cell infiltration between the high and low risk groups. **(B)** The correlation between the signature genes and immune cells. **(C)** TumorPurity between the two risk groups. **(D)** ESTIMATEScore between the two risk groups. **(E)** ImmuneScore between the two risk groups. **(F)** StromalScore between the two risk groups. **(G)** Comparison of the scores of TIDE between the high and low risk group. *<0.05; ns, Not Significant.

The CIBERSORT algorithm was used to calculate the immune cell infiltration between the high and low risk groups, and the results showed that Plasma cells, T cells CD4 memory activated, Macrophages M1 and Mast cells activated were significantly higher in the high-risk group, while T cells CD4 memory resting, T cells regulatory (Tregs) Dendritic cells activated, Mast cells resting and Eosinophils were higher in the low-risk group (**Table S4**) (**Figure 13A**). Also, we analyzed the correlation between the signature genes and immune cells (**Figure 13B**).

### 3.11 Tumor microenvironment and immunotherapy analysis

**Figure 14:**
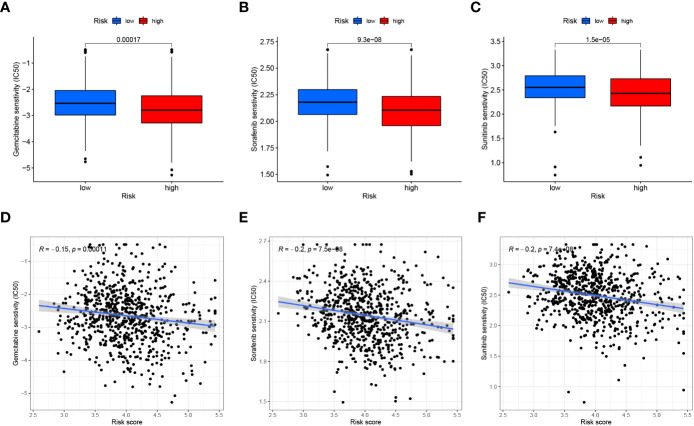
Drug sensitivity analysis. **(A–C)** Comparison of the Gemcitabine, Sorafenib and Sunitinib (IC50) between the two groups. **(D–F)** The relationship between risk score and Gemcitabine, Sorafenib and Sunitinib (IC50) sensitivity.

Similarly, we assessed the ImmuneScore, StromalScore, ESTIMATEScore and TumorPurity levels between the two risk groups using the ESTIMATE method, and the results showed that the tumor purity was higher and the non-tumor components were lower in the high-risk group (**Figures 13C–F**). To compare the differences in immunotherapy between the two risk groups, we analyzed the risk of immune escape between the two groups, and the results showed that the high-risk group had less risk of immune escape and might respond better to immunotherapy (**Figure 13G**). We also performed an analysis of risk score versus drug sensitivity, and the results showed that the high-risk group was more sensitive to Gemcitabine, Sorafenib and Sunitinib and may have better treatment outcomes. Correlation analysis showed that the higher the risk score, the higher the sensitivity to these three drugs (**Figures 14A–F**).

## 4 Discussion

HCC is one of the most common malignancies worldwide and its morbidity and mortality are on the rise (18, 19). Surgery remains the most successful treatment modality available, but most HCC usually occurs in the setting of cirrhosis, where the postoperative residual liver has poor hepatic function while still maintaining a tumor-prone microenvironmental state. The possibility of cure exists through radiofrequency ablation and liver transplantation, but requires diagnosis and intervention at a sufficiently early stage. In this context, the advent of immune checkpoint inhibitors (ICIs), tyrosine kinase inhibitors (TKIs) and monoclonal antibodies has expanded the therapeutic field for HCC. Immunotherapy is currently a major hotspot in the treatment of HCC, and understanding the interplay between oncogenic pathways and immune responses is essential to improve current and future therapeutic outcomes.

The pathogenic role of organelles in tumors has received increasing attention, and the Golgi apparatus complex is a highly dynamic organelle that is considered the “heart” of intracellular transport. In the last decade, a growing number of studies have reported structural alterations of the Golgi apparatus associated with diseases, dysregulation and infection. These include stress responses (20), cancer (21) and various infections. The mammalian GA is the center of glycosylation and changes in GA structure will lead to reordering of glycosyltransferases, resulting in specific glycosylation epitopes that lead to tumorigenesis and development (22). In addition, GA membrane dynamics are triggered by the actin cytoskeleton and associated unconventional myosin (23). In many cases, upregulation of GA associated myosin motility is associated with aggressive cancers (24, 25). Meanwhile, myosin 18 is able to coordinate directly with GA morphology by interacting with Golgi phosphorylated protein 3 (GOLPH3), and their linkage triggers Golgi proliferation (26). In turn, the synergistic GOLPH3-myosin 18a relationship is necessary for DNA damage to induce Golgi fragmentation, which itself is a prerequisite for most mutations and cancers (27).

Due to the important role of the GA in tumor development, this study utilized bioinformatics analysis techniques based on the study of GARGs to identify two distinct molecular subtypes that exhibit significantly different immune cell infiltration and survival outcomes by comprehensive mining of publicly available HCC transcriptional data. Meanwhile, a risk score signature consisting of three GARGs was constructed using these key GARGs to predict the prognosis and immunotherapy response in HCC patients. The C2 cluster and low risk score groups in this study exhibited abundant immune cell infiltration and more non-tumor cell components, along with lower tumor purity, characteristics that corresponded to better survival outcomes. Meanwhile, the high-risk group exhibited a lower risk of immune escape and higher sensitivity to immunotherapeutic agents, findings that provide new perspectives for improving patient prognosis and risk-stratified treatment strategies by considering TME characteristics and transcriptomics.

Among the three genes that constitute the prognostic signature, RNF24 is considered as one of the risk factors for human oral squamous cell carcinoma (28). Moreover, RNF24 expression is more than 2-fold upregulated in esophageal adenocarcinoma patients compared to normal subjects (29). BSG (CD147) is a multifunctional protein involved in cancer cell survival and controls lactate transport mainly through interaction with monocarboxylate transporter proteins (MCT) such as MCT1 (30). It was shown that CD147 receptor is essential for TFF3-mediated signaling that regulates colorectal cancer progression (31). In addition, CD147 is able to promote collective invasion of hepatocellular carcinoma cells through upregulation of histone B expression (32). kIF20A (kinesin family member 20A), was shown to promote proliferation and metastasis of bladder cancer cells, and bladder cancer patients with high expression of KIF20A had poorer tumor differentiation and poorer prognosis (33). Moreover, overexpression of KIF20A can promote tumor proliferation and invasion in renal clear cell carcinoma and is associated with poor clinical outcome (34). In our study, a nomogram was constructed to calculate a score representing the OS of HCC patients through a risk scoring system composed of these three genes. The calibration plots showed that the signature had a better fitting curve, better clinical application than the traditional staging system, and was able to predict the prognosis of HCC patients more individually. These three GARGs have been studied and identified to have regulatory roles in tumors, thus providing higher value for our signature to predict prognosis and immunotherapeutic response in hepatocellular carcinoma.

## 5 Conclusion

In summary, we have systematically explored for the first time the value of GARGs in the prognosis and immunotherapy of patients with HCC, providing a new direction and therapeutic target for the treatment of HCC.

## Data availability statement

Publicly available datasets were analyzed in this study, the names of the repositories/accession numbers are listed within the article/**Supplementary Material**.

## Ethics statement

The studies involving human participants were reviewed and approved by the Ethics Committee of the Second Affiliated Hospital of Nanchang University. The patients/participants provided their written informed consent to participate in this study. The animal study was reviewed and approved by the Ethics Committee of the Second Affiliated Hospital of Nanchang University.

## Author contributions

LS, ZL and ZheW designed the study and performed the experiments and wrote the manuscript. KN, JH, ZC and ZhiW assisted with specimen collection and bioinformatics analysis. XY reviewed the article and made revisions. All authors contributed to the article and approved the submitted version.
