# Supplementary material for: Molecular subtype identification and signature construction based on Golgi apparatus-related genes for better prediction prognosis and immunotherapy response in hepatocellular carcinoma
Source: Front Immunol. 2023 Mar 27;14:1113455. doi: 10.3389/fimmu.2023.1113455 (PMC10083374; doi:10.3389/fimmu.2023.1113455)
Supplement: Supplementary file 1 [file DataSheet_1.docx]

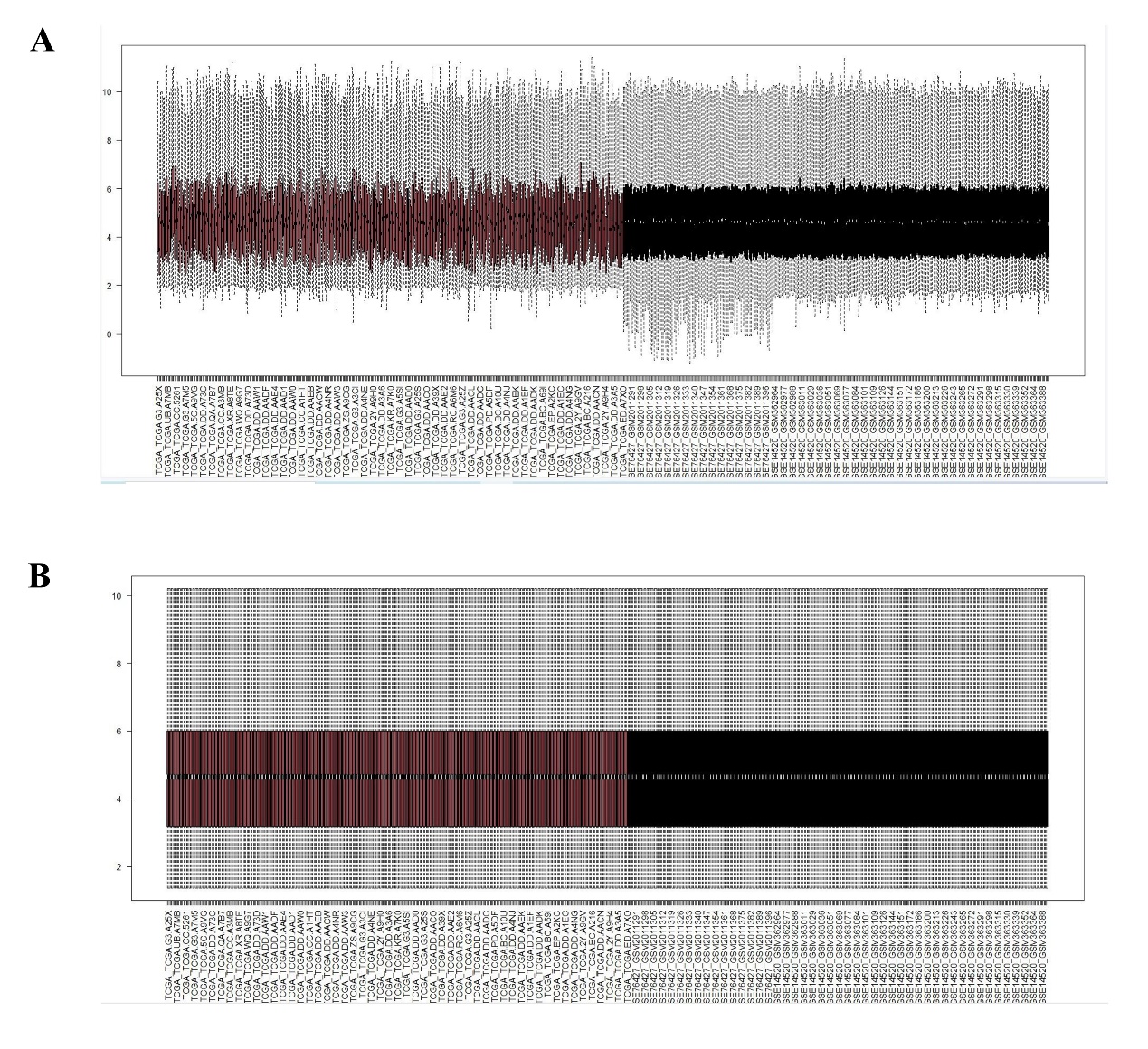


**Figure S1 | Batch correction and standardization of three data sets. (A)** Batch correction of three data sets. **(B)** Data standardization.
