# Supplementary figures and images for: Molecular subtype identification and signature construction based on Golgi apparatus-related genes for better prediction prognosis and immunotherapy response in hepatocellular carcinoma
Source: Front Immunol. 2023 Mar 27;14:1113455. doi: 10.3389/fimmu.2023.1113455 (PMC10083374; doi:10.3389/fimmu.2023.1113455)

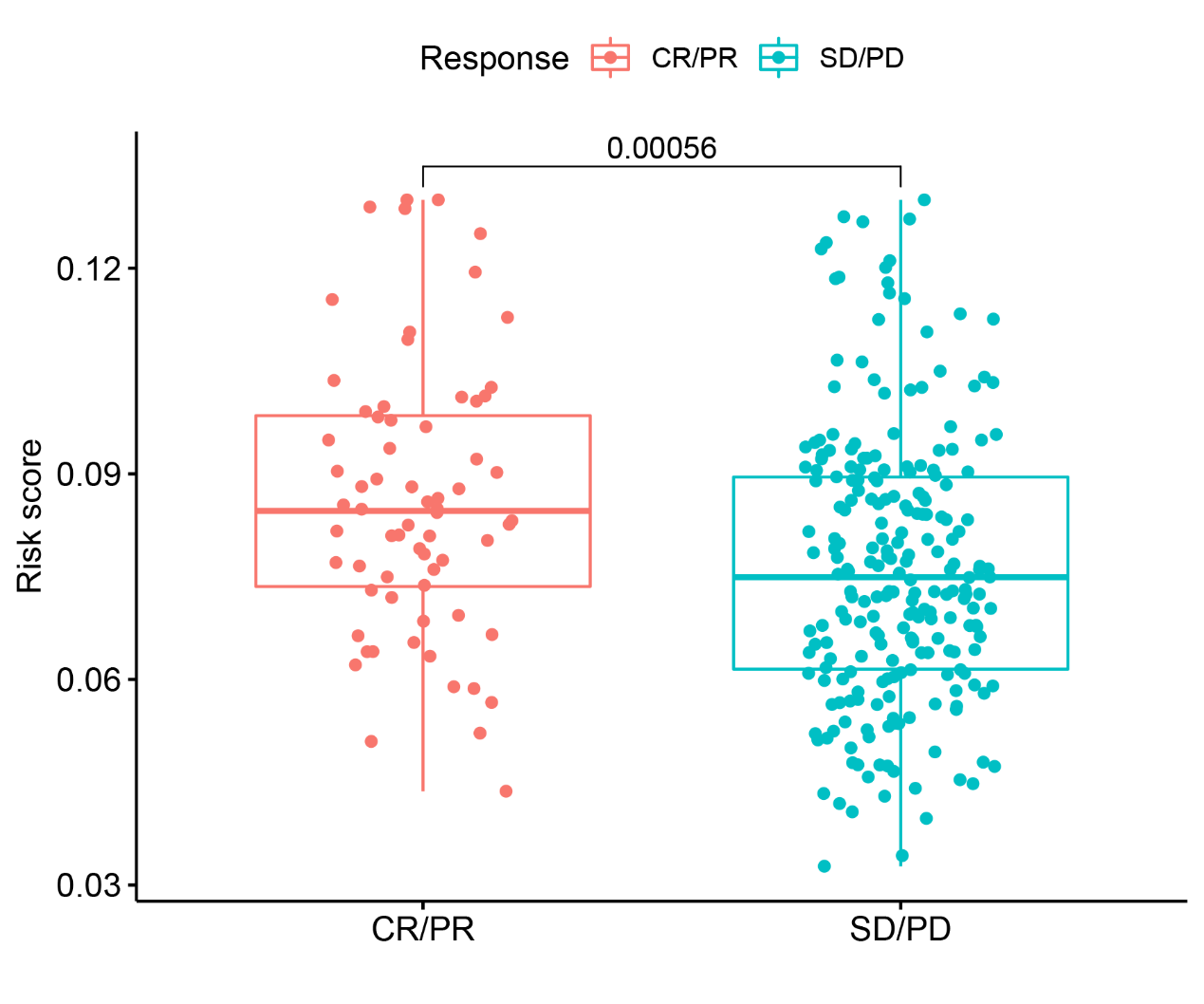


**Figure S2 | Immunotherapy response.**

Supplement: Supplementary file 2 [file DataSheet_2.docx]
