# Supplementary material for: Molecular subtype identification and signature construction based on Golgi apparatus-related genes for better prediction prognosis and immunotherapy response in hepatocellular carcinoma
Source: Front Immunol. 2023 Mar 27;14:1113455. doi: 10.3389/fimmu.2023.1113455 (PMC10083374; doi:10.3389/fimmu.2023.1113455)
Supplement: Supplementary file 3 [file DataSheet_3.docx]

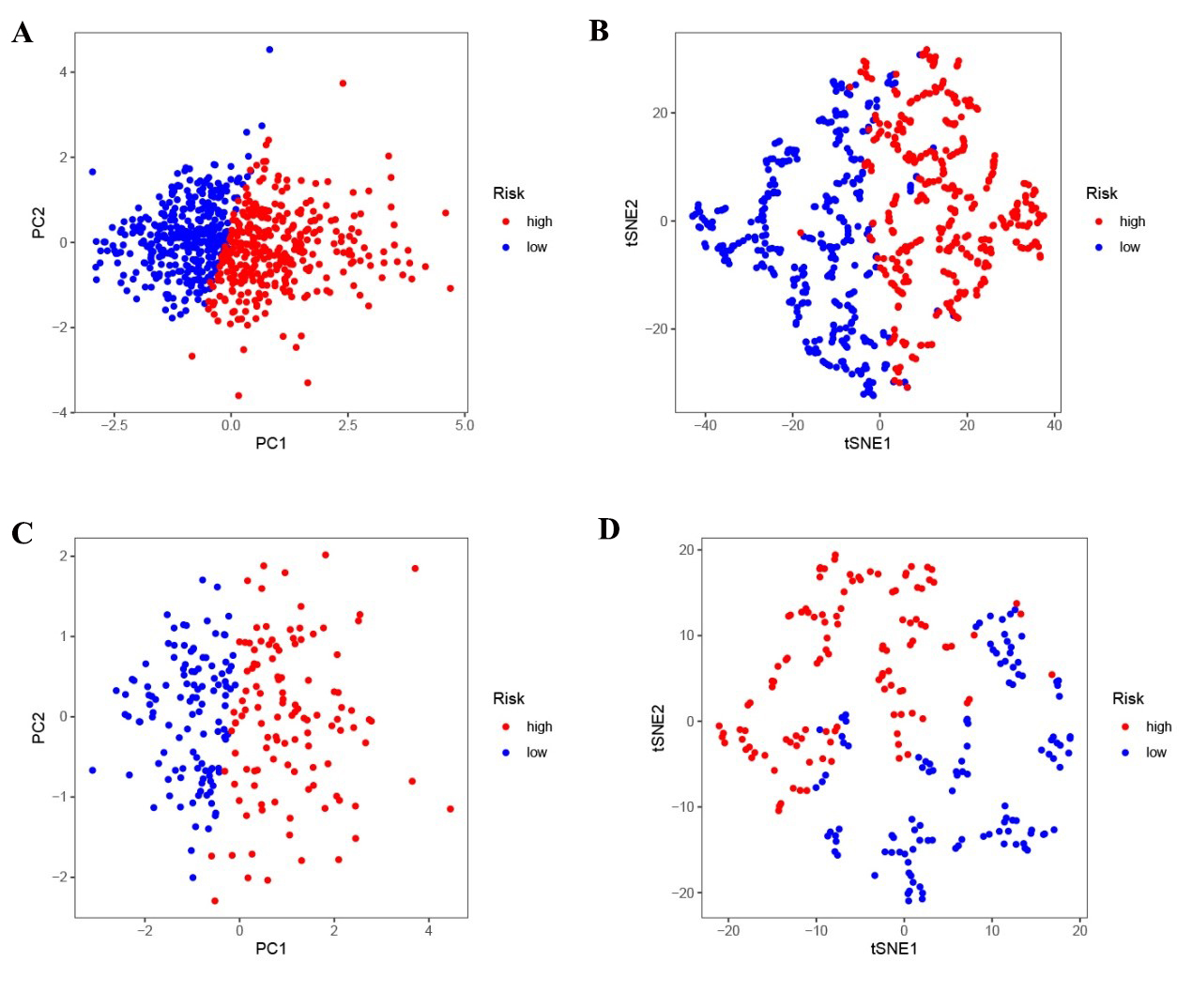


**Figure S3 | t-SNE and PCA between the high and low risk groups in TCGA and ICGC. (A)** PCA analysis in TCGA. **(B)** t-SNE in TCGA. **(C)** PCA analysis in ICGC. **(D)** t-SNE in ICGC.
