# Supplementary material for: Molecular subtype identification and signature construction based on Golgi apparatus-related genes for better prediction prognosis and immunotherapy response in hepatocellular carcinoma
Source: Front Immunol. 2023 Mar 27;14:1113455. doi: 10.3389/fimmu.2023.1113455 (PMC10083374; doi:10.3389/fimmu.2023.1113455)
Supplement: Supplementary file 4 [file DataSheet_4.docx]

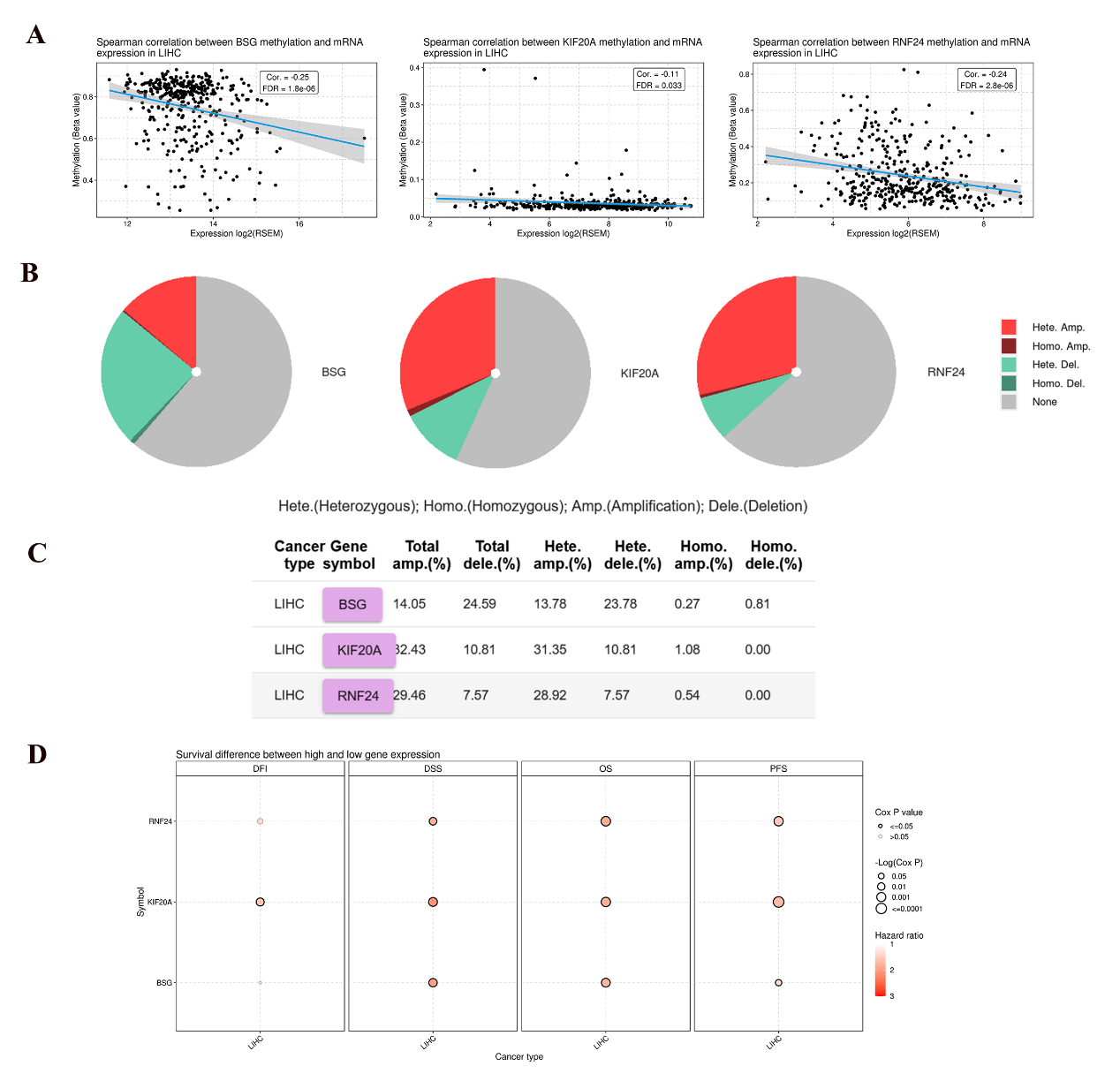


**Figure S4 | The methylation levels and copy number variation of the signature genes in LIHC using the GSCA database.** **(A)** Correlation between the expression of the three signature genes and the degree of methylation. **(B, C)** Copy number variation frequency of 3 signature genes in LIHC. **(D)** Analysis of survival differences in 3 signature genes in LIHC.
